# High Arterial Glucose is Associated with Poor Pressure Autoregulation, High Cerebral Lactate/Pyruvate Ratio and Poor Outcome Following Traumatic Brain Injury

**DOI:** 10.1007/s12028-019-00743-2

**Published:** 2019-05-23

**Authors:** Teodor Svedung Wettervik, Timothy Howells, Elisabeth Ronne-Engström, Lars Hillered, Anders Lewén, Per Enblad, Elham Rostami

**Affiliations:** Department of Neuroscience, Section of Neurosurgery, Uppsala University, 751 85 Uppsala, Sweden

**Keywords:** Traumatic brain injury, Glucose, Autoregulation, Cerebral energy metabolism

## Abstract

**Background:**

Arterial hyperglycemia is associated with poor outcome in traumatic brain injury (TBI), but the pathophysiology is not completely understood. Previous preclinical and clinical studies have indicated that arterial glucose worsens pressure autoregulation. The aim of this study was to evaluate the relationship of arterial glucose to both pressure reactivity and cerebral energy metabolism.

**Method:**

This retrospective study was based on 120 patients with severe TBI treated at the Uppsala University hospital, Sweden, 2008–2018. Data from cerebral microdialysis (glucose, pyruvate, and lactate), arterial glucose, and pressure reactivity index (PRx55-15) were analyzed the first 3 days post-injury.

**Results:**

High arterial glucose was associated with poor outcome/Glasgow Outcome Scale-Extended at 6-month follow-up (*r* = − 0.201, *p* value = 0.004) and showed a positive correlation with both PRx55-15 (*r* = 0.308, *p* = 0.001) and cerebral lactate/pyruvate ratio (LPR) days 1–3 (*r* = 0. 244, *p* = 0.014). Cerebral lactate-to-pyruvate ratio and PRx55-15 had a positive association day 2 (*r* = 0.219, *p* = 0.048). Multivariate linear regression analysis showed that high arterial glucose predicted poor pressure autoregulation on days 1 and 2.

**Conclusions:**

High arterial glucose was associated with poor outcome, poor pressure autoregulation, and cerebral energy metabolic disturbances. The latter two suggest a pathophysiological mechanism for the negative effect of arterial hyperglycemia, although further studies are needed to elucidate if the correlations are causal or confounded by other factors.

## Introduction

Traumatic brain injury (TBI) is the leading cause of mortality and morbidity in young adults [1]. Management of TBI aims at reducing secondary brain injuries by maintaining physiological parameters such as intracranial pressure (ICP), mean arterial blood pressure (MAP), cerebral perfusion pressure (CPP), and arterial euglycemia.

Arterial hyperglycemia is common post-TBI and has been associated with poor outcome [2, 3]. This may in part be due to a negative effect on pressure autoregulation, as preclinical studies have shown that arterial hyperglycemia decreases cerebral and systemic blood flow and reduces endothelial function, indicating worsened autoregulation [4–7]. Pressure autoregulation is the capacity to maintain an adequate, unchanged cerebral blood flow (CBF) over a wide range of CPPs. This is an important mechanism to avoid cerebral hypo-/hyperperfusion that may lead to cerebral ischemia or edema, respectively. Pressure autoregulation may be measured continuously as the pressure reactivity index (PRx), that is, the correlation coefficient between changes in MAP and ICP [8]. A negative PRx indicates preserved pressure autoregulation, i.e., an increase in MAP leads to vasoconstriction to maintain CBF which in turn leads to less cerebral blood volume and ICP [8]. Pressure autoregulation may become deranged post-TBI with high PRx values, which has been associated with worse outcome [9, 10]. Endothelial, myogenic, and metabolic factors have been suggested to disturb the cerebral autoregulation, but the exact pathophysiological mechanisms remain unclear [11]. In a recent clinical study by Donnelly et al., PRx had a positive, significant correlation with arterial glucose after TBI [12]. Young et al. had similar findings in a pediatric TBI population [13]. However, the interaction between arterial glucose, PRx, and cerebral energy metabolism has not been elucidated.

The aim of this study was to further investigate the association between arterial glucose, pressure autoregulation and cerebral energy metabolism to better understand the pathophysiological mechanisms of secondary brain injuries in TBI.

## Materials and Methods

### Patients and Study Design

Patients with severe TBI were admitted to the Department of Neurosurgery at the University Hospital in Uppsala, Sweden. Out of 1001 TBI patients (2008–2018), there were 120 patients that met the study inclusion criteria, i.e., neurointensive care (NIC) with intubation, mechanical ventilation and monitoring of arterial blood pressure, ICP, and cerebral energy metabolism (cerebral microdialysis). Patients were treated in accordance with our standardized ICP-oriented treatment protocol to avoid secondary insults [14]. Treatment goals were ICP ≤ 20 mm Hg, CPP ≥ 60 mm Hg, systolic blood pressure > 100 mm Hg, central venous pressure 0–5 mm Hg, pO_2_ > 12 kPa, arterial glucose 5–10 mmol/L (mM), electrolytes within normal ranges, normovolemia, and body temperature < 38 °C. The arterial glucose was monitored with repeated arterial blood gases. Hyperglycemia was corrected with short-acting insulin injections and in some cases intravenous insulin infusions for patients with diabetes mellitus.

### Data Acquisition and Analysis

ICP was monitored with either an intraparenchymal (Codman ICP Micro-Sensor, Codman & Shurtleff, Raynham, MA) or an intraventricular catheter drainage system (HanniSet, Xtrans, Smith Medical GmbH, Grasbrunn, Germany). Arterial blood pressure was measured invasively in the radial artery. Arterial blood gas (ABG) data were generally analyzed from the radial arterial line every fourth hour, more often if needed. PRx, traditionally calculated as the 5 min correlation of 10 s averages of ICP and MAP, was used in combination with a bandpass filter, limiting the analysis to oscillations with periods of 15–55 s (PRx55-15). It has previously been shown that PRx55-15 has better signal-to-noise ratio and correlation with outcome than the traditional PRx [9].

Cerebral energy metabolism was monitored with the 71 high cut-off microdialysis analyzer (MD) catheter with a membrane length of 10 mm and a membrane cut-off of 100 kDa (M Dialysis AB, Stockholm, Sweden). The catheters were perfused by means of a microinjection pump (106 MD Pump, M Dialysis AB) at a rate of 0.3 µL/min with custom-made sterile artificial cerebrospinal fluid (CSF) containing—NaCl 147 mmol/L (mM), KCl 2.7 mM, CaCl_2_ 1.2 mM, MgCl_2_ 0.85 mM, and 1.5% human albumin. Cerebral extracellular glucose, lactate, pyruvate, and urea were measured hourly, using a CMA 600 analyzer or the ISCUSflex Microdialysis Analyzer (M Dialysis AB). The MD urea was monitored to validate catheter performance [15]. The MD was placed in normal-appearing brain tissue in the right frontal lobe, adjacent to the ICP monitor.

### Outcome

Clinical outcome was assessed at 6-month post-injury, by specially trained personnel with structured telephone interviews, using the Glasgow Outcome Scale-Extended (GOS-E), containing eight categories of global outcome, from death to upper good recovery [16–18].

### Statistical Analysis

Mean daily (24 h) values were calculated for each patient the first 3 days and for the entire 3-day-period (72 h) post-injury including ICP (mm Hg), CPP (mm Hg), PRx55-15, arterial glucose (mM), cerebral glucose (mM), cerebral lactate (mM), cerebral pyruvate (µM), and cerebral lactate-to-pyruvate ratio (LPR) in our software Odin [19]. The mean daily (24 h) and 3-day-period (72 h) values of ICP, CPP, and PRx55-15 values were calculated using minute-by-minute data. The corresponding mean arterial glucose values were based on ABG analyses from approximately every fourth hour. The mean values of cerebral glucose, cerebral pyruvate, and cerebral LPR were based on hourly analyses. All data were transferred to SPSS version 25 (IBM Corp, Armonk, NY, USA) for further statistical analysis. The Shapiro–Wilk test was used to test the normal distribution assumption. PRx55-15 showed normal distribution at all time intervals except day 2, whereas arterial glucose, cerebral glucose, cerebral pyruvate, cerebral lactate, cerebral LPR, and GOS-E did not show normal distribution. Hence, the Spearman correlation test was used to investigate the association between mean daily values of arterial glucose, PRx55-15, cerebral glucose, cerebral pyruvate, cerebral lactate, cerebral LPR, and outcome. There were only a few missing observations and those were excluded from the analyses. A *p* value < 0.05 was considered statistically significant. Many of the correlation tests were dependent, such as arterial glucose versus pyruvate, lactate and LPR, and correction tests which are particularly conservative in this case. As this was an exploratory study with preclinical support, we did not adjust for multiple comparisons, to avoid type II-errors. A multivariate linear regression analysis was done in the first subsequent 3 days post-injury to predict PRx55-15 based on age, Glasgow Coma Scale motor (GCS M) at admission, and mean ICP, CPP, and arterial glucose for each day.

### Ethics

All procedures performed in the studies involving humans were in accordance with the ethical standards of the institutional and national research committee and with the 1964 Helsinki declaration and its later amendments or comparable ethical standards. Informed consent was obtained by the relatives of all participating patients.

## Results

### Demographic Data

There were 120 TBI patients included in this study. The mean age was 43 (± 20) years and 75% were male (Table 1). Median GCS M was 5 (interquartile range (IQR) 4–5). Outcome data showed a mortality rate of 10% and median GOS-E was 5 (IQR 3–7).

**Table 1:** Demographic data

Patient, *n*	120
Age (years), mean (± SD)	43 (± 20)
Sex (male), *n* (%)	90 (75%)
GCS M at admission, median (IQR)	5 (4–5)
GOS-E, median (IQR)	5 (3–7)
Mortality, *n* (%)	12 (10%)

*GCS M* Glasgow Coma Scale motor, *GOS-E* Glasgow Outcome Scale-Extended, *IQR* interquartile range, *SD* standard deviation

### Description of Neurophysiological Parameters the First 3 Days Post-TBI

The mean ICP was 11 (± 8.5) mm Hg and the mean CPP was 75 (± 12) mm Hg on the first day post-injury (Table 2). The pressure autoregulatory status (PRx55-15) was worse on the first day of trauma with a mean value at 0.22 (± 0.21), but gradually improved to 0.16 (± 0.22) on the third day.

**Table 2:** Mean values for neurophysiological and arterial blood gas parameters

	Day 1	Day 2	Day 3	Days 1–3
ICP, mean (± SD) mm Hg	11 (± 8.5)	13 (± 10)	14 (± 12)	13 (± 10)
CPP, mean (± SD) mm Hg	75 (± 12)	73 (± 12)	74 (± 14)	74 (± 12)
Arterial glucose, mean (± SD) mM	8.8 (± 2.1)	8.4 (± 2.3)	8.0 (± 1.3)	8.3 (± 1.3)
PRx55-15, mean (± SD) coefficient	0.22 (± 0.21)	0.19 (± 0.18)	0.16 (± 0.22)	0.17 (± 0.18)
Cerebral glucose, mean (± SD) mM	2.6 (± 1.3)	2.2 (± 1.1)	2.1 (± 1.1)	2.2 (± 1.0)
Cerebral lactate, mean (± SD) mM	3.6 (± 2.5)	3.6 (± 2.3)	3.7 (± 2.2)	3.6 (± 2.2)
Cerebral pyruvate, mean (± SD) µM	144 (± 90)	137 (± 52)	144 (± 52)	142 (± 51)
Cerebral LPR, mean (± SD)	27 (± 20)	29 (± 28)	36 (± 86)	27 (± 22)

*CPP* cerebral perfusion pressure, *ICP* intracranial pressure, *LPR* lactate/pyruvate ratio, *PRx55-15* pressure reactivity index, *SD* standard deviation

The mean arterial glucose was 8.8 (± 2.1) mM on the first day of trauma and decreased the consecutive 2 days, to 8.0 (± 1.3) mM on day three. The percentage of blood gases with hypoglycemic events, defined as arterial glucose below 5 mM, was at the mean value 1.7 (± 7.1) % on the first and 0.98 (± 5.6) % on the third day post-injury. The percentage of blood gases with hyperglycemic events, defined as arterial glucose above 10 mM, was at the mean value 19 (± 27) % on the first day post-injury and the mean value was 6.6 (± 15) % on day three.

**Fig. 1:**
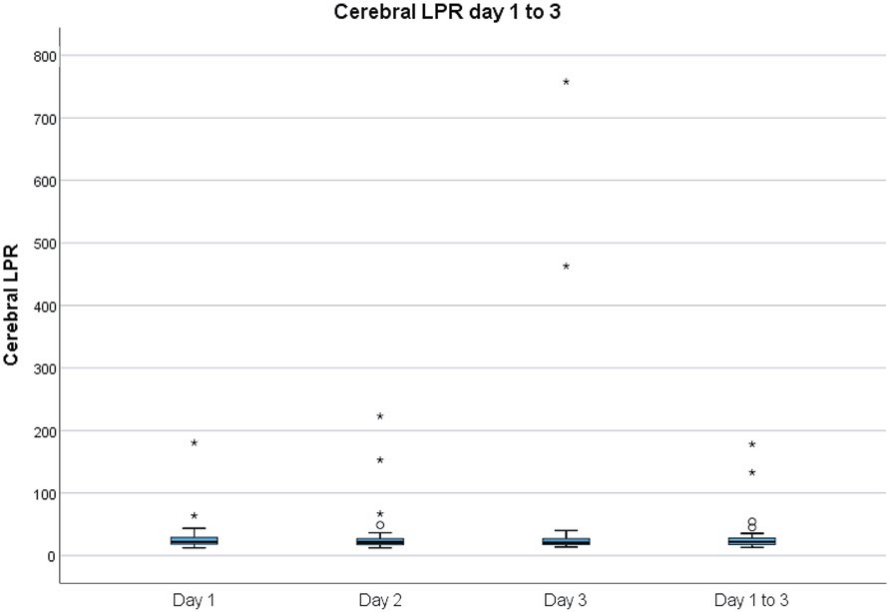
Cerebral LPR days 1–3 post-TBI. The figure shows a box-plot of the distribution of cerebral LPR on the first 3 days post-injury. The circle and star indicate outliers and extreme outliers, respectively

The mean cerebral glucose decreased to some extent from 2.6 (± 1.3) to 2.1 (± 1.1) mM from days 1 to 3. The cerebral lactate was stable over the first 3 days, the mean value was 3.6 (± 2.5) mM on the first day of trauma. The cerebral pyruvate was 144 (± 90) µM on day 1 and 144 (± 52) µM on day 3 post-injury. The mean cerebral LPR gradually increased from 27 (± 20) on days 1 to 36 (± 86) on day 3. One patient had a LPR above 100 on day 1 (LPR = 181), three patients on day 2 (LPR = 134, 152, and 223, respectively), and two patients on day 3 (LPR = 463 and 758, respectively) (Fig. 1). The patients with high LPR on day 3 had developed intracranial hypertension above 50 mm Hg. There were no negative values; the standard deviation was high due to these outliers.

Sixty-eight of the 120 patients had at least one LPR value above 25 on the first 3 days. The cause of the pathologically high LPR was considered ischemic, when the concurrent cerebral pyruvate was below the ischemic threshold at 120 µM [20, 21], otherwise more indicative of a mitochondrial dysfunction. Cerebral pyruvate was below the ischemic threshold 120 µM in median 33 (IQR 0–84) % of the times LPR was above 25 in these 68 patients. Hence, cerebral pyruvate was more commonly normal to high at times of metabolic disturbances with LPR above 25, indicating mitochondrial dysfunction in these cases.

### Relationship Among Arterial Glucose, Injury Severity, Neurophysiological Parameters, and Cerebral Metabolites

**Fig. 2:**
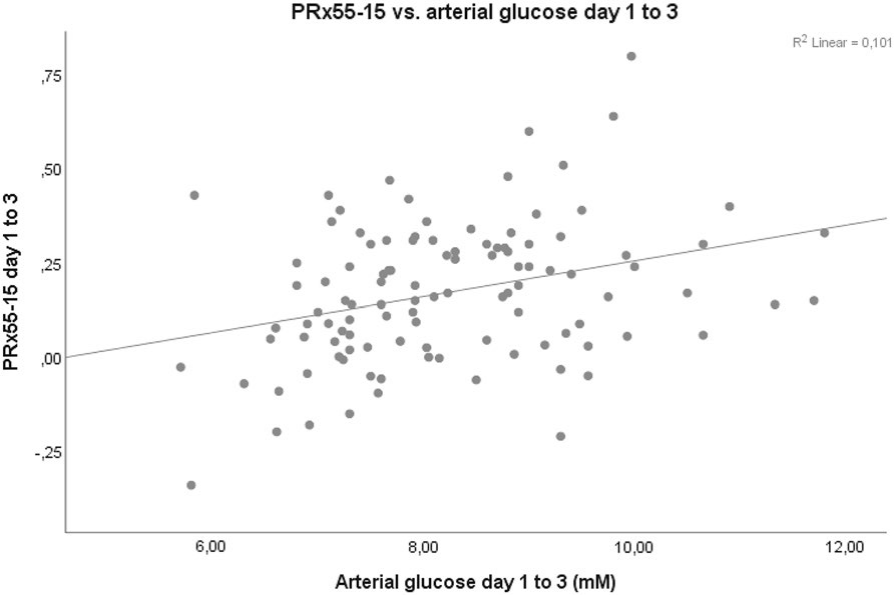
PRx55-15 versus arterial glucose days 1–3 post-TBI. The figure shows the correlation between mean PRx55-15 and arterial glucose days 1–3 post-TBI (*r* = 0.308, *p* = 0.001)

**Fig. 3:**
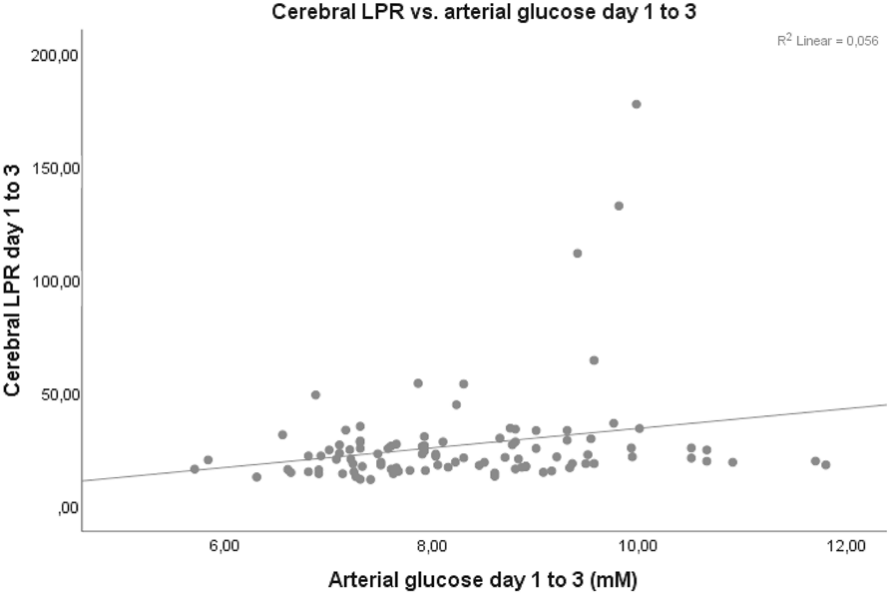
Cerebral LPR versus arterial glucose days 1–3 post-TBI. The figure shows the correlation between mean cerebral LPR and arterial glucose days 1–3 post-TBI (*r* = 0.244, *p* = 0.014)

**Fig. 4:**
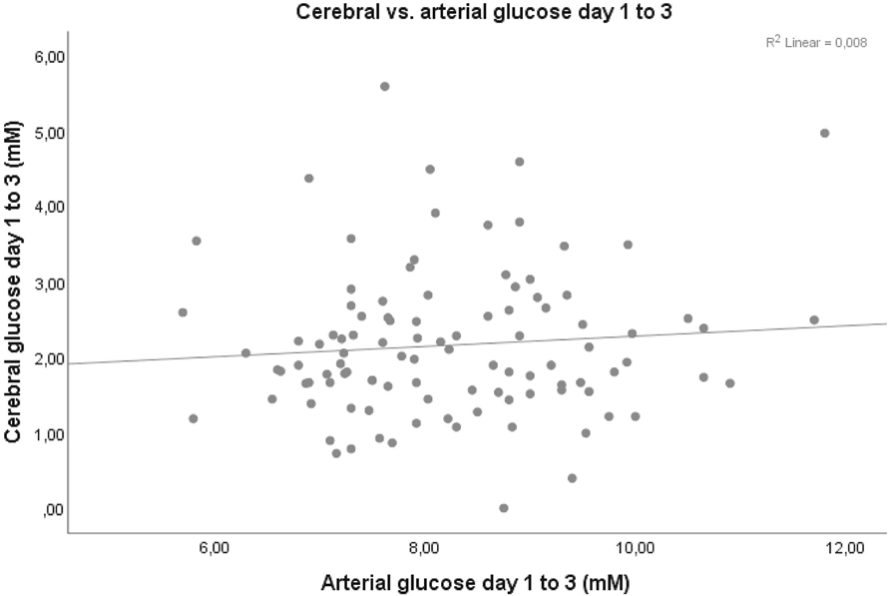
Cerebral versus arterial glucose days 1–3 post-TBI. The figure shows the correlation between mean cerebral and arterial glucose days 1–3 post-TBI (*r* = 0.071, *p* = 0.479)

Arterial glucose and GCS M at admission did not correlate significantly on any of the first 3 days post-injury. However, mean arterial glucose correlated positively with mean PRx55-15 days 1 to 3 (*r* = 0.308, *p* value = 0.001), i.e., high arterial glucose with high PRx55-15/disturbed pressure reactivity (Fig. 2). Arterial glucose and PRx55-15 also had positive correlations with *p* values < 0.05 on days 2 and 3 (Table 3). Arterial glucose and cerebral LPR were associated days 1, 2 and 1–3 with *p* values < 0.05 (Fig. 3). Cerebral LPR and PRx55-15 had a positive association day 2 (*r* = 0.219, *p* = 0.048). Cerebral glucose (Fig. 4), cerebral pyruvate, and cerebral lactate had no correlation with PRx55-15 nor arterial glucose.

**Table 3:** Relation between arterial glucose, PRx55-15 and cerebral metabolism (Spearman)

	Day 1	Day 2	Day 3	Days 1–3
Arterial glucose versus cerebral glucose (*r*)	0.240	0.114	0.126	0.071
*p* value	0.072	0.294	0.223	0.479
Arterial glucose versus cerebral pyruvate (*r*)	0.036	0.042	0.010	0.063
*p* value	0.790	0.697	0.920	0.527
Arterial glucose versus cerebral lactate (*r*)	0.096	0.205	0.072	0.151
*p* value	0.479	0.056	0.480	0.129
Arterial glucose versus cerebral LPR (*r*)	0.268	0.287	0.139	0.244
*p* value	0.050	0.008	0.182	0.014
Arterial glucose versus PRx55-15 (*r*)	0.129	0.298	0.259	0.308
*p* value	0.315	0.004	0.007	0.001
PRx55-15 versus cerebral glucose (*r*)	− 0.174	− 0.011	0.176	0.114
*p* value	0.196	0.918	0.092	0.254
PRx55-15 versus cerebral pyruvate (*r*)	0.067	− 0.064	0.064	0.086
*p* value	0.620	0.555	0.538	0.392
PRx55-15 versus cerebral lactate (*r*)	0.167	0.132	0.146	0.163
*p* value	0.215	0.224	0.159	0.102
PRx55-15 versus cerebral LPR (*r*)	0.245	0.219	0.093	0.148
*p* value	0.077	0.048	0.382	0.148

*LPR* lactate/pyruvate ratio, *PRx55-15* pressure reactivity index

Four multivariate linear regression analyses were done to predict PRx55-15 on each of the first 3 days and the entire 3-day-period post-injury (Table 4). All analyses included age, GCS M at admission and the mean ICP, CPP, and arterial glucose of the day. Arterial glucose was a significant predictor of PRx55-15 on both days 1 and 2, but not on day 3. Increasing values of arterial glucose predicted higher PRx55-15/worse autoregulation these first 2 days. Increasing age predicted poor pressure autoregulation on day 3, and increasing ICP predicted worse pressure autoregulation on both days 2 and 3. Exclusion of those two patients that developed intracranial hypertension above 50 mm Hg attenuated ICP to be only a marginally significant predictor of PRx55-15, but did not otherwise have any major impact on the other coefficients.

**Table 4:** Prediction of PRx55-15 the first 3 days post-injury—a multiple linear regression analysis

	Day 1		Day 2		Day 3		Days 1–3	
	SC	*p* value	SC	*p* value	SC	*p* value	SC	*p* value
Age (years)	− 0.15	0.31	0.066	0.56	0.26	0.012	0.20	0.073
GCS M	0.024	0.85	− 0.027	0.78	− 0.087	0.34	− 0.043	0.63
ICP (mm Hg)	0.29	0.12	0.39	0.011	0.51	0.004	0.44	0.006
CPP (mm Hg)	− 0.10	0.59	− 0.091	0.54	0.14	0.42	0.013	0.93
Arterial glucose (mM)	0.29	0.041	0.25	0.026	0.024	0.81	0.14	0.21

The table shows the multiple linear regression analysis to predict PRx55-15. *R*^2^ day 1 = 0.25, ANOVA, *p* value = 0.005. *R*^2^ day 2 = 0.33, ANOVA, *p* value < 0.001. *R*^2^ day 3 = 0.29, ANOVA, *p* value < 0.001. *R*^2^ days 1–3 = 0.30, ANOVA, *p* value < 0.001

*CPP* cerebral perfusion pressure, *GCS M* Glasgow Coma Scale motor, *ICP* intracranial pressure, *SC* standardized coefficient

### Clinical Outcome Versus Neurophysiological Parameters and Cerebral Metabolites

Clinical outcome, defined as GOS-E, correlated significantly with the mean values of PRx55-15 days 1–3 (*r* = − 0.389, *p* value < 0.001), i.e., high PRx55-15 was associated with poor outcome (low GOS-E) (Table 5). There was also a negative association between arterial glucose days 1–3 and GOS-E (*r* = − 0.201, *p* value = 0.004), i.e., high arterial glucose with poor outcome (low GOS-E). The correlation between mean arterial glucose day 1 with GOS-E decreased from (*r* = − 0.381, *p* = 0.001) to (*r* = − 0.203, *p* = 0.045) on day 3 post-injury. The percentage of ABG with hyperglycemia (arterial glucose > 10 mM) only correlated with poor outcome on day 1 post-injury (*r* = − 0.251, *r* = 0.036). No associations could be detected between cerebral glucose, cerebral pyruvate, cerebral lactate, cerebral LPR, and clinical outcome.

**Table 5:** Correlation between clinical outcome at 6 months and physiological parameters

GOS-E versus	*r*	*p* value
PRx55-15 days 1–3	− 0.389	< 0.001
Arterial glucose days 1–3	− 0.201	0.004
Cerebral glucose days 1–3	− 0.131	0.200
Cerebral pyruvate days 1–3	− 0.081	0.428
Cerebral lactate days 1–3	− 0.108	0.292
Cerebral LPR days 1–3	− 0.108	0.302

*GOS-E* Glasgow Outcome Scale-Extended, *LPR* lactate/pyruvate ratio, *PRx55-15* pressure reactivity index

## Discussion

In the current study, the main results were that high arterial glucose was associated with poor pressure autoregulation, high LPR and worse clinical outcome. However, the cerebral LPR only correlated weakly on day 2 with pressure autoregulation. Otherwise, LPR disturbances were mostly characterized by high lactate and normal to high pyruvate, indicating mitochondrial dysfunction. This suggests that arterial glucose and LPR may interact via metabolic rather than cerebrovascular pathways.

In line with prior studies [13, 22], we found high arterial glucose to be associated with poor outcome (Table 4). As the mean daily arterial glucose decreased from days 1 to 3, the association between arterial glucose and outcome decreased in a similar way. However, mean arterial glucose had a stronger correlation with clinical outcome than the percentage of hyperglycemic insults (arterial glucose > 10 mM), indicating that also subthreshold values of arterial hyperglycemia may be deleterious for outcome. Several preclinical studies have demonstrated the worsening of a cerebral ischemic injury by hyperglycemia, particularly in stroke models [23]. The pathophysiology of high arterial glucose in TBI is not fully elucidated; however, according to preclinical studies, arterial hyperglycemia decreases blood flow in systemic and cerebral blood vessels and causes endothelial dysfunction, indicating a link with failed cerebral autoregulation [4–7]. Two clinical studies have shown a weak, significant correlation between pressure autoregulation and arterial glucose in adult and pediatric TBI, respectively [12, 13], consistent with our results in the current study.

In contrast to these earlier studies, we also evaluated cerebral MD parameters and found an association between high arterial glucose with high cerebral LPR (Table 3). The lactate/pyruvate ratio describes the cellular redox state and high values indicate energy metabolic disturbances, which are correlated with brain tissue damage and poor outcome in TBI [24, 25]. High arterial glucose was associated with both poor pressure autoregulation and high LPR days 1–3, but PRx55-15 showed significant correlation with LPR only on day 2. As previously demonstrated, cerebral hyperemia is common [26] and poor pressure autoregulation predicts poor outcome particularly well on day 2 post-injury [27]. This may explain why the link between the pressure autoregulatory status (PRx55-15) and brain tissue damage (LPR) was present only on day 2.

As the energy metabolic disturbances with LPR above 25 were most likely due to mitochondrial dysfunction in our study, it is possible that pathophysiological metabolic pathways of arterial glucose were more important than cerebrovascular dysregulation for these cerebral energy disturbances. For example, arterial hyperglycemia may generate mitochondrial dysfunction and cause energy crisis in neurons [28]. In this study, we used cerebral pyruvate below 120 µM to distinguish between ischemia and mitochondrial dysfunction in cases of metabolic disturbances with LPR above 25. However, brain tissue oxygenation could have further improved this dichotomization. Future studies combining CBF measurements, brain oxygen monitoring, PRx55-15, and MD could shed better light on this pathophysiology.

It is uncertain if the correlations between arterial glucose with pressure autoregulation and cerebral LPR are causal or secondary to confounding factors such as primary injury severity and catecholamine release [29, 30]. For example, arterial hyperglycemia has been shown to correlate with poor outcome in cases of post-traumatic stress, but not in cases due to comorbid diabetes mellitus [31]. It is necessary to determine the influence of catecholamines and vasopressors on arterial glucose, pressure autoregulation and clinical outcome. In this study, only six patients had concurrent diabetes mellitus, which is why no further subgroup analysis was done. However, high arterial glucose independently predicted poor pressure autoregulation days 1 and 2, even after adjustment for injury severity (GCS M) and other factors such as age, ICP, and CPP.

Both low [32] and high [25] cerebral glucose levels have been shown to correlate with poor outcome and it has been widely debated how arterial glucose should be managed to optimize the cerebral glucose levels and to what degree the arterial and cerebral glucose levels correlate the following TBI. Magnoni et al. showed a preserved, positive, linear correlation between arterial and cerebral glucose in TBI [33]. However, we have earlier found that this correlation varied over time post-injury and was only preserved in the uninjured parts of the brain [34]. Diaz-Parejo also found a positive correlation between arterial and cerebral glucose, but cerebral lactate was only increased in cases of arterial hyperglycemia above 15 mM [35]. Although our study had a larger study population compared with the other studies, we found no correlation between mean arterial and cerebral glucose on any of the first 3 days post-TBI (Fig. 3). One explanation could be our NIC-unit protocol with an attentive nurse with frequent ABG analyses that prohibited episodes of severe hypo- or hyperglycemia.

Furthermore, arterial glucose, but not focal cerebral glucose, correlated with the pressure autoregulatory status. This may be explained by that PRx55-15 is a global measure of the cerebrovascular reactivity, and arterial glucose is similarly a global measure of the glucose concentration in the cerebral arterial vessels. On the contrary, the cerebral glucose measure represents a focal brain region with weaker correlation to the global state.

The paradox of arterial glucose management in TBI is that whereas hyperglycemia per se is associated with poor outcome and deranged neurophysiology, tight glycemic control with intensive insulin therapy (IIT) management to normoglycemia has failed to reduce mortality and improve outcome in TBI [36–39]. There were some advantages with IIT such as shorter intensive care unit stay and lower infection rate, but it also led to an increased frequency of hypoglycemia. Furthermore, IIT post-TBI generated worse MD parameters with lower cerebral glucose and increased cerebral LPR than conventional glycemic control [22, 39]. Critically, low interstitial cerebral glucose commonly observed the following TBI, e.g., in conjunction with spreading depolarizations, has been associated with unfavorable outcome [40]. Due to the lack of clear evidence of benefits for IIT in the neurointensive care setting, a more loose glycemic control is mostly applied [23]. This indicates that we still know little about the pathophysiology and optimal glucose management in TBI. Further insights into the pathophysiology of hyperglycemia on, e.g., cerebrovascular reactivity and cerebral metabolism over time post-TBI may lead to better arterial glucose management protocols that can improve outcome.

### Limitations

Limitations of the study include the following. First, the number of significance tests was plenty, increasing the risk for type I errors, without adjustment for multiple corrections. However, this was as hypothesis generating study and many correlations such as arterial glucose versus PRx55-15 were highly significant (*p* value = 0.001). Furthermore, although arterial glucose correlated significantly with PRx55-15 and LPR, the coefficients were relatively weak. This indicates that other factors than arterial glucose are important for the pressure autoregulatory status and cerebral energy metabolism.

## Conclusions

Arterial hyperglycemia correlates with poor outcome in TBI, but the mechanism is unclear. We have shown that high arterial glucose was associated with poor outcome and disturbed cerebral pressure autoregulation, consistent with preclinical studies indicating adverse effects of hyperglycemia on cerebrovascular function and CBF. High arterial glucose was also associated with cerebral metabolic disturbances post-TBI, as indicated by high LPR. There was also an indication that during the second day post-injury, the effect of disturbed autoregulation on the metabolic state of the injured brain is more pronounced. There is a need to better evaluate if there is a causal nature of these correlations with arterial glucose. Insights into these relationships may result in better arterial glucose management and improved patient outcome.
